# What Enables Novel Thoughts? The Temporal Structure of Associations and Its Relationship to Divergent Thinking

**DOI:** 10.3389/fpsyg.2018.01771

**Published:** 2018-09-25

**Authors:** Peng Wang, Maarten L. Wijnants, Simone M. Ritter

**Affiliations:** Behavioural Science Institute, Radboud University Nijmegen, Nijmegen, Netherlands

**Keywords:** creativity, divergent thinking, associations, LSA, semantic distance, complex systems, temporal structure, interdisciplinary

## Abstract

The aim of the current study is to enhance our understanding of cognitive creativity, specifically divergent thinking, by employing an interdisciplinary methodological approach. By integrating methodology from computational linguistics and complex systems into creativity research, the current study aims to shed light on the relationship between divergent thinking and the temporal structure of semantic associations. In complex systems, temporal structures can be described on a continuum from random to flexible-stable and to persistent. Random structures are highly unpredictable, persistent structures are highly predictable, and flexible-stable structures are in-between, they are partly predictable from previous observations. Temporal structures of associations that are random (e.g., dog–graveyard–north pole) or persistent (e.g., dog–cat–rat) are hypothesized to be detrimental to divergent thinking. However, a flexible-stable structure (e.g., dog–police–drugs) is hypothesized to be related to enhanced divergent thinking (inverted-U). This notion was tested (*N* = 59) in an association chain task, combined with a frequently used measure of divergent thinking (i.e., Alternative Uses Test). Latent Semantic Analysis from computational linguistics was used to quantify the associations, and methods from complex systems in form of Power Spectral Density analysis and detrended fluctuation analysis were used to estimate the temporal structure of those associations. Although the current study does not confirm that a flexible-stable (vs. random/persistent) temporal structure of associations is related to enhanced divergent thinking skills, it hopefully challenges fellow researchers to refine the recent methodological developments for assessing the (temporal) structure of associations. Moreover, the current cross-fertilization of methodological approaches may inspire creativity researchers to take advantage of other fields’ ideas and methods. To derive a theoretically sound cognitive theory of creativity, it is important to integrate research ideas and empirical methods from a variety of disciplines.

## Introduction

Creativity has often been defined as the generation of novel and useful insights or solutions to a problem (e.g., Stein, 1953; Runco and Jaeger, 2012). However, the question of what creativity really should be, is rather complex. Some scholars have offered conceptual frameworks that capture a wide scope of many research directions that entail creativity. One example is the Four C Model of creativity by Kaufman and Beghetto (2009, 2013) where a distinction between mini-c, little-c, pro-c, and big-c is made. This separation of four levels of creativity is mainly driven by the indirect assumption of different gradients of experiences. Therefore, mini-c focuses on developmental and transformative experiences in children and little-c more on everyday life accomplishments. For example, a child that learns to tie their shoes in a different way solved a problem in a new manner. This accomplishment wouldn’t be regarded as ‘creative’ for an adult in their daily life routine. Pro-c, on the other hand, distinguishes accomplishments in professional settings that are transformative for certain arts or crafts (e.g., inventing a new statistical method) but is lacking the eminent accomplishment that revolutionizes the world (e.g., formulating probability theory). Those eminent accomplishments could be understood as big-c following Kaufman and Beghetto. Consequently, the Four C Model helps to embed different creative outputs in settings that are hardly comparable to another. Research assessing little-c creativity has gained considerable knowledge to this date. For example, creativity is found to be linked to intelligence, in that creative potential benefits from intelligence (or vice versa). There is evidence that creativity might benefit from intelligence up to a certain level but not above that level (e.g., Jauk et al., 2013; Karwowski and Gralewski, 2013). Others argue that intelligence might be necessary but not sufficient for creative potential and that this relationship does not stringently follow a curvilinear shape (e.g., Karwowski et al., 2016). Further, there is evidence that attentional flexibility is linked to creative potential (e.g., Zabelina et al., 2015a,b), that creativity can be trained (e.g., Scott et al., 2004; Ritter and Mostert, 2017), and that positive and negative mood moderate creative thought differently (e.g., De Dreu et al., 2008). What has remained relatively unexplored are the cognitive underpinnings and foundations of creativity. It has been proposed that a core ability in the process of generating creative solutions involves divergent thinking, which refers to the process of producing multiple answers to a problem (Guilford, 1967; Plucker et al., 2011). Divergent thinking, in turn, is believed to rely on the ability to generate remote semantic associations (Mednick, 1962; Levin, 1978; Acar and Runco, 2014). A semantic association–in this study–is the written lexical response (e.g., door) to another lexical stimuli (e.g., house) (Osipow and Grooms, 1966). Thus far, little research has been conducted to uncover the possible mechanism that allows people to express divergent thinking. Semantic associations are proposed to contribute to divergent thinking (e.g., Acar and Runco, 2014).

In a recent special issue of the *Journal of Creative Behavior*, celebrating its 50^th^ anniversary, it has been argued that creativity research would benefit from more interdisciplinary work, encompassing different perspectives (Ambrose, 2017). In the current study, we combined *creativity research* on divergent thinking with methodology from *computational linguistics* and *complex systems*. Computational linguistics approaches phenomena in language from a computational perspective, utilizing statistical models (Manning and Schütze, 1999). Complex systems, briefly, is the study of how parts in a system interact to create behavior that cannot be explained by examining the parts alone (i.e., an holistic approach; Bassingthwaighte et al., 1994). The outline of the introduction is as follows: First, an introduction to divergent thinking and association formation with respect to creativity is presented. Second, ideas from complex systems are discussed in light of association formation in creativity. Third, techniques from computational linguistics, such as Latent Semantic Analysis (LSA), and in particular semantic distance (SmD) are introduced as a measurement technique for studying creativity. LSA has been increasingly applied in creativity research recently (e.g., Green, 2016; Hass, 2017b; Forthmann et al., 2018).

### The Role of Associations in Divergent Thinking

Since Guilford (1950) introduced the idea of divergent and convergent thinking, these concepts have been very prominent in creativity research. While convergent thinking is defined as the process of finding one single, correct solution to a problem, the notion of divergent thinking is regarded as the opposite process. Divergent thinking relies on the generation of various solutions to a problem (Cropley, 2006). A widely used method to measure divergent thinking is the Alternative Uses Test (AUT; Guilford, 1950; Kaufman et al., 2008). In this test, participants are asked to generate as many ideas as possible about the usage of a commonplace object (i.e., “What can you do with a brick?”). A typical scoring scheme incorporates creativity (i.e., perceived creativity of the ideas generated), novelty (i.e., originality of the ideas), usefulness (i.e., applicability of ideas), and fluency (i.e., the number of ideas generated).

An influential theoretical model advanced by Mednick (1962) proposes that remote associations play a vital role in the formation of creative ideas. For example, Benedek et al. (2012) assessed dissociative ability and associative combination as different associative abilities with regard to divergent thinking. Dissociative ability reflects the ability to form unrelated concepts from previous concepts (e.g., summer: computer, bridge,…). Associative combination refers to the ability to form reasonable associations to seemingly unrelated concepts (e.g., summer–high: airplane, temperature,…). The authors found that dissociative ability and associative combination predicted divergent thinking, which was a composite of fluency and originality. Another interesting study from Forthmann et al. (2016) used eight different AUTs and the authors manipulated the instructions and the lemma frequencies of the objects in the tasks. That is, there was one condition that received a to “be creative” instruction while the other condition should focus on fluency. Lemma frequency or word stem frequency was varied across the objects in the AUTs so that some objects would have more natural occurrences in language and others less. Results indicated that objects with a high lemma frequency also led to more generated ideas. The interaction between instruction and lemma frequency on the fluency of ideas revealed that objects with high frequency evoked less ideas in the “be creative” condition compared to the other one. For low frequency objects the fluency of ideas was similar in both conditions. Those findings showcase that associations play a different role depending on the task-relevant goals in the instructions.

According to Mednick (1962), individual differences in creative abilities are due to differences in their hierarchy of associations. More creative individuals should possess a “flatter” associative hierarchy, in which the strength of the associations is more similar to each other, whereas less creative individuals would show a “steeper” hierarchy. To illustrate this with an example, a highly creative individual would respond to the word “dog” with more unusual associations (e.g., work, police, and drugs) than less creative individuals (e.g., cat, pet, and bird). For a highly creative individual, distinct concepts are more closely related, in that the concept “dog–drugs” has the same associative strength as “dog–cat.” On the other hand, a less creative individual has a much stronger association between “dog” and “cat,” and a rather weak association between “dog” and “drugs.” Hence, highly creative individuals form a “flatter” associative hierarchy (dog–police = dog–cat = dog–drugs …), whereas it is suggested that less creative individuals have a “steeper” associative hierarchy (dog–cat > dog–police > dog–drugs …). Consequently, highly creative individuals should be able to access remote associates with more ease, to ultimately form a creative solution. In a similar vein, Rossmann and Fink (2010) tested students with higher creativity-related demands (i.e., enrolled in an art college) and students with lower creativity-related demands (i.e., enrolled in psychology and geosciences) in a word-pair task. The participants were instructed to judge the associative distance between indirectly related word pairs (e.g., cat–cheese) and unrelated word pairs (e.g., subject–marriage) on a 6-point scale. Results of this study indicated that students with higher creativity-related demands, compared to students with lower creativity-related demands, estimated the associative distance between unrelated word-pairs as lower and hence, more proximate to each other.

After reviewing inconclusive studies, Benedek and Neubauer (2013) conducted an experiment to test Mednick’s assumption. The authors used a continuous free association task in which associations had to be created for six predefined words within 60 s per word. Subsequently, participants were categorized as high or low creatives based on their performance on two other divergent thinking tasks (i.e., unrelated to the free association task). The results indicated that individuals scoring high on creativity, as measured by the two divergent thinking tasks, also formed more associations and more uncommon responses. The authors could not find evidence that corroborates differences in associative hierarchy and concluded that hierarchy does not contribute to divergent thinking. However, there is ample evidence suggesting that, for example, the semantic networks of high versus low creative people inherit different properties. Kenett et al. (2014, 2016) used methodologies from network science to test Mednick’s hypothesis and found that highly creative people have a denser, less modular (less sub-parts) and more connected semantic network. Hence, high creatives are supposed to access remote associations more efficiently, with more interconnections and shorter routes between two or more concepts.

We discussed that associations may play a vital role in divergent thinking. Further, it has been theorized that the structure of those associations is crucial for divergent thinking (Mednick, 1962) and there are indications that properties of semantic networks might be related to divergent thinking (e.g., Kenett et al., 2014). To further explore the theorized associative structure underlying divergent thinking, this study investigates the temporal structure of associations. Temporal structure, in this case, refers to the change of associations over time (e.g., dog–cat–milk–supermarket…). In other words, how are differently organized “chains of thoughts,” which unfold over time, related to divergent thinking? It is hypothesized that different temporal structures of associations underlie the ability to perform well in divergent thinking. This idea will be elaborated in more detail after complex systems and SmD have been introduced. In the following part, ideas and methodologies from complex systems will be discussed.

### Complex Systems

Complex systems are mainly embraced in mathematics and natural sciences to describe processes that originate in nature. What characterizes these systems are their non-linear, dynamical and mutual influential, and interdependent properties (Friedenberg, 2009). The core assumption is that knowledge about only one part of a system will not lead to knowledge about the overarching behavior of the system. Consider for example the case of population swings in a predator–prey model as an illustration (Berryman, 1992). Let there be a population of foxes (predator) and a population of rabbits (prey) interacting in an environment. If we observe both population changes over time, the number of rabbits and foxes will vary in a non-linear way. It will be the case that the number of rabbits decreases if the number of foxes increases. However, if there are very few rabbits left, the population of foxes will decline dramatically as there is not enough prey. This is accompanied by a minor increase of rabbits at first, which is then followed by an exponential grow of rabbits. By gaining knowledge about only the rabbits (physiology and ethology) or only the foxes, one cannot explain the change in population over time. In order to understand the change in population (either rabbits, foxes, or both), the whole interacting system has to be observed. This example illustrates that as little as two variables or parts of a system can produce behavior that is neither linear, nor independent, as these two parts of the system mutually interact.

**FIGURE 1:**
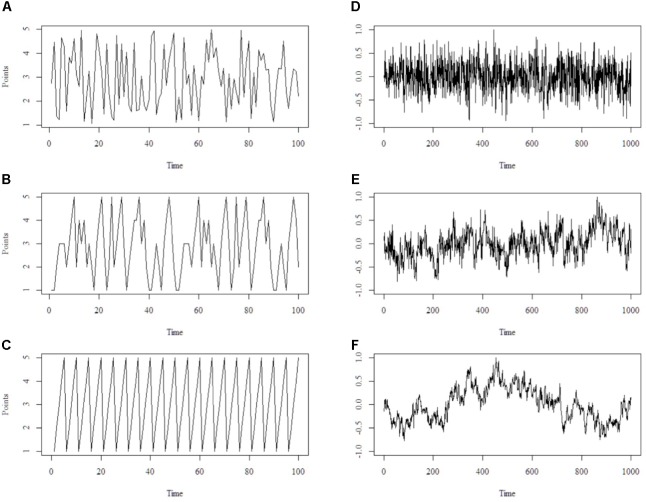
Schematic illustration of the outcomes of the ball-throw game on the left, points are displayed on the y-axis and time on the x-axis. Throw pattern of player A is random **(A)**, throw pattern of player B is flexible-stable **(B)** and throw pattern of player C is persistent **(C)**. On the right, three different time-series that correspond to random pattern **(D)**, flexible pattern **(E)** and persistent pattern **(F)** are shown. Those time-series reflect self-similarity across different scales. That is, the pattern of **(E)** observed over 1000 trials (bigger scale) is already reflected in, for example, the first 200 trials (smaller scale) or trials 400 to 800 (intermediate scale). It is self-similar as e.g., the first 200 trials of **(E)** are resembling the whole time-series of **(E)**. Put differently, the first 200 trials of **(E)** “look” similar to trial 0–500 or 0–1000 of **(E)**. Notice that not only the geometrical shape but also statistical properties are similar across scales.

Another crucial aspect is the temporal order of observations in a complex system. The temporal order is important as parts of a complex system are mutually influencing one another (which changes over time). An analogy will exemplify the reasoning behind this idea. Assume we test three players in a skill game where they throw a tennis ball in one of five bowls, representing 1–5 points. Each player has a different skill level/technique and throws for 100 trials. We call these 100 trials a *time-series*. Player A randomly hits the bowls (e.g., instruct a computer to generate a throwing scheme). Player A ends up with an average of 3 points across all trials. Player C would display a very persistent throwing order. He would decide to hit the bowls in an ascending order (e.g., hit bowl 1, then 2, then 3,…), repeating the pattern until he reaches 100 trials. Thus, he would be very persistent in his throws, but his average would also be 3 points. Player B is flexibly varying his throws in that he sometimes hits one bowl more often, and then switches. However, he is neither random nor very persistent in his actions, so that the pattern of throwing is not obvious at first glance. He also ends up with an average of 3 points. Further, all three players would deviate from the mean by approximately 1.4 points. With classical frequentist statistics, we would not be able to discern the underlying difference between the three players. Data would be pooled and the temporal structure of the time-series was lost. It is apparent that the temporal order is substantially different between the three players (see **Figure 1**, right panel). The order or strategy of player A (random) would substantially differ from player C (very persistent) or B (flexible-stable). Therefore, the temporal structure or dynamics of the time-series should be taken into account to capture the whole picture. Models from complex systems adhere to the temporal structure in a time-series and preserve them in the analysis.

Mathematically, the temporal structure of time-series can be distinguished into at least three classes of patterns. Those patterns are typically called either random, persistent or flexible-stable (Wijnants, 2014). Notice that this is a continuum, where randomness is the one extreme and persistence the other, with flexible-stability residing in the middle balancing random and persistent processes out. Processes in a time-series constitute change over time, they reflect what happened at time 1, time 2, time 3, and so on. A time-series can be regarded as random when the next observation (e.g., time 4) can hardly be predicted from the previous data (e.g., time 1–3). Thus, it does not build on previous observations. A time-series can be regarded as persistent when the next observation (e.g., time 4) can be determined with high certainty from the previous data. Thus, it strongly builds on previous observations. A time-series can be regarded as flexible-stable when the next observation (e.g., time 4) can be determined with moderate certainty. That is, higher than random and lower than persistent. Thus, it does partly build on previous observations but can vary to some extent. Linking to the previously mentioned ball game, each player’s time-series could be described in the continuum from random (i.e., player A) to flexible-stable (i.e., player B) to persistent (i.e., player C).

#### Complex Systems in Physiological and Psychological Research

Research indicates that the functioning of humans also obeys temporal regularities. Physiological examples show that the time-series of performances on a motor task is associated with flexible-stable patterns (Wijnants et al., 2009) and likewise does a healthy heartbeat fluctuation reflect a flexible-stable pattern (Van Orden et al., 2011) whereas abnormal fluctuations will not (Peng et al., 1995; Goldberger, 1997). Studies on cognitive processes show that the reaction-time of mental rotation (Gilden and Hancock, 2007), word naming and simple reaction tasks (Van Orden et al., 2003) are associated with flexible-stable patterns. Accordingly, it has been proposed that those processes which lie in between persistent and random can be regarded as optimal (Corona et al., 2013).

Interestingly, it has been argued that complex systems might be a promising approach to study creativity. For example, Piccardo (2017) examined the potential role of plurilingualism and the associated dynamical engagement with one’s language and environment as a beneficial factor in creative thought. Further, Poutanen (2013) suggests that different levels of inquiries (i.e., individual level, group level and organizational level) about creative phenomena can be embedded within a complex systems framework. The present study investigates a time-series reflecting a chain of associations. Put differently, it examines how associations unfold and change over time. By implementing methodology from a complex systems perspective, the temporal structure of this chain of associations can be inferred. Distinctions can be attributed to a chain or structure of associations to discern random, flexible-stable or persistent patterns. In order to utilize this approach, a time-series is needed. In the following part of the introduction, we will address the possibility to quantify a time-series from a chain of written associations.

### Computational Linguistics, Latent Semantic Analysis, and Semantic Distance

One approach in computational linguistics involves the quantification of word similarities using large amounts of texts. This line of reasoning relies on the distributional hypothesis, stating that words with similar meaning tend to occur together in a similar context (Harris, 1954; Sahlgren, 2008). A prominent method for modeling word similarities is LSA (Deerwester et al., 1990; Landauer and Dumais, 1997). LSA is a computational method that allows the user to compress substantial quantity of texts and retrieve their word meanings or semantics. It does so by creating a highly dimensional spatial space (*semantic space*) where semantic concepts are represented as points (vectors) in this space. Subsequently, it is possible to infer the relative position between, for example, two words in this semantic space by calculating the cosine (number between 0 and 1) of the angle of those two points. For instance, there are studies showing that the similarity between words, expressed by the cosine in LSA, significantly predicts reaction time in lexical priming experiments (e.g., Jones et al., 2006; Hutchison et al., 2008; Günther et al., 2016).

Recently, researchers in the field of creativity have started to measure creative performance by using LSA to estimate the similarity between words as SmD (e.g., Green et al., 2006; Beaty et al., 2014). SmD is now defined as the inverted cosine (1-cosine) of two semantic concepts (two points or words in the semantic space), where higher decimals represent more dissimilarity and lower decimals represent more similarity between two concepts. Hence, the higher the SmD of two concepts, the less common they are, and vice versa. For example, the concept of “university” and the concept of “cook” do not share much common ground (SmD of 0.87, greater distance and lower similarity). If you now compare “university” to “study,” the SmD drops as those concepts are more related to each other and appear together more often (SmD of 0.44, smaller distance and higher similarity).

#### Evidence and Validity of Semantic Distance as a Measure for Creativity

In an extensive study by Hass (2017a), responses from two AUTs were quantified using LSA. The author found, for example, that the SmD from the responses, directed to the target concept (i.e., brick and bottle), was non-linearly increasing as the task progresses. As a result, this means that participants generated associations more closely related to the target concept at the beginning but that this relatedness slowly decays as the process continues. Similarly, per-trial response time was positively correlated with SmD between adjacent responses and creativity scores provided by raters. In another paper by the same author, a large data set with divergent thinking tasks was reanalyzed with LSA. As in the previous study, responses from different AUTs were first quantified using LSA and then analyzed in a regression model. The results indicated that subjective creativity ratings were positively predicted by SmD. That is, more dissimilar concepts or ideas of the AUTs were associated with more creative ratings (Hass, 2017b). Another line of evidence comes from neuropsychological studies. For example, Green et al. (2010) manipulated SmD of word pairings and measured (left) frontopolar cortex activity. The (left) frontopolar cortex is believed to be involved in the process of analogical reasoning, which plays a vital role in innovative outcomes. The authors showed that a higher SmD was associated with greater frontopolar cortex activity. In another study by the same group of researchers (Green et al., 2015), participants were now tested in an analogy generation task. Here, a verb had to be generated to a noun shown to the participants. Crucially, half of the trials were cued, signalizing that a creative verb should be formed. Explicit instruction to think creatively has previously been shown to be effective in enhancing creative performance (e.g., Runco and Okuda, 1991; Chen et al., 2005; Hong et al., 2016). Results indicated that SmD was significantly higher for cued trials than for uncued trials. Further, for cued trials, the (left) frontopolar cortex exhibited increased activity. As a direct extension to those results, stimulating the frontopolar cortex through transcranial Direct Current Stimulation has been observed to enhance performance in the same cued analogy task (Green et al., 2016). SmD has also been used directly as an outcome variable for creativity in a study testing a large online sample (Weinberger et al., 2016). Here, an analogy finding task was used and the authors were able to find that SmD was higher for blocks that were paired with the instruction to think creatively than without the instruction.

Finally, construct validity for SmD as a measure for creativity was provided by Prabhakaran et al. (2014). The authors showed that SmD positively correlates with creativity measurements, specifically with a divergent thinking task, story writing task, the Torrance figural test and the Creative Achievement Questionnaire. Based on these findings, SmD has been suggested as a novel measurement tool to reliably measure creativity. Other work on the reliability of LSA in creativity research has been done by Forthmann et al. (2018) who revisited several studies which applied LSA to quantify responses from divergent thinking tasks. They argued that estimations of SmDs are potentially biased due to response length (i.e., multiple words) and conducted a simulation study. When responses were removed from stop words and corrected for biases, the authors found the correlation between SmD and creativity ratings in divergent thinking tasks to be highest.

In the present study LSA and SmD is utilized to quantify a time-series of a chain of associations. Hereby, the change over time in the similarity of a chain of associations can be inferred by implementing methodologies from complex systems. That is, a persistent pattern in a chain of associations would reflect similar concepts that build closely on previous concepts (e.g., dog–cat–milk–cow, etc., hence, all low SmD). A random pattern would reflect extremely dissimilar concepts that are loosely related to previous concepts (e.g., dog–graveyard–north pole–whisky–etc., hence, all high SmD). Lastly, a flexible-stable pattern would reflect dissimilar concepts that build on previous concepts (e.g., dog–police–helicopter–fan–etc., hence, intermediate SmD).

### The Present Study

It was discussed that the ability to generate associations plays a vital role in divergent thinking. That is, associative abilities (e.g., combining distinct associations) contribute to divergent thinking (Benedek et al., 2012). Further, Mednick (1962) suggested that the associative hierarchy is crucial, which received support from studies involving semantic networks (Kenett et al., 2014; but for an exception, see Benedek and Neubauer, 2013). This study proposes that the temporal structure of associations is related to divergent thinking. Temporal structure here refers to the change of generated (written) associations over time. By applying methodologies from complex systems, a time-series can be characterized on a continuum from random to persistent. LSA and SmD will be used to quantify a testable time-series, where the association between two concepts serves as observations that unfold over time. More precisely, it is hypothesized that a flexible-stable (in between random and persistent) temporal structure of associations is linked to the highest performance on the creativity and originality dimension of divergent thinking. It is assumed that associations that are too random would make less sense and are therefore regarded as less creative (e.g., from “dog” to arbitrary concepts like “graveyard”). On the other hand, too persistent associations do not create novel and creative insights as they too strongly rely on previous associations (e.g., from “dog” to “cat”). In the middle, where flexible-stable associations are found, it is expected that concepts are unrelated enough to create novel and thus creative insights (e.g., “dog” and “drugs”).

Hence, novelty and creativity in divergent thinking are hypothesized to be predicted by a quadratic term (or inverted U) of the temporal structure of associations. In a novel paradigm, where a chain of associations had to be formed, SmD was calculated using LSA. Subsequently, the temporal structure was inferred through Power Spectral Density (PSD) analysis and detrended fluctuation analysis (DFA, described in the Methods section). The Brick Version of the AUT served as a measure for divergent thinking. Additionally, it is expected to observe a positive relationship between mean SmD, originality and creativity in the divergent thinking task. This would add to previous findings on the validity of SmD as a creativity measure (e.g., Green, 2016) To complement the analysis, a convergent thinking task and a real-life creative achievement questionnaire are added for explorative analysis.

## Materials and Methods

### Participants

Participants were 59 students (41 female) from the Radboud University Nijmegen with a mean age of 21.95 years (*SD* = 2.58). All participants were of German nationality and spoke German on a native level. Participation was voluntary and rewarded with either €7.50 or credit points, which were to be obtained as part of the participants’ curriculum. One participant was excluded from the descriptive and correlation analysis (*N* = 58), however, the main analysis (*N* = 59) included all participants. Exclusion was based on unreasonable scores on the CAQ (87, highest score for all categories, fulfilled several times). Descriptive scores are shown in **Table 1**.

**Table 1:** Descriptive statistics of creativity measures and PSD, DFA.

Variable	*M*	*SD*	Range
AUT			
Creativity	2.29	0.44	1.38–3.79
Novelty	2.43	0.48	1.38–3.79
Usefulness	3.51	0.57	1.61–4.50
Fluency	7.47	3.19	2–16
CRAT	4.27	2.59	0–11
CAQ	5.10	2.60	0–11
PSD	-1.24	0.48	-2.49 – -0.26
DFA	0.54	0.08	–0.39–0.79

*N = 58 for CAQ statistics as one participant was excluded due to an unreasonable CAQ score of 87. N = 59 for all other variables*.

### Divergent Thinking, the Alternative Uses Test (AUT)

A widely used test for divergent thinking is the AUT (Guilford, 1950; Runco and Acar, 2012). The brick version of the AUT was used in the present study and was introduced as an idea generation task to the question: “What can you do with a Brick?–List your ideas below”^1^. The task was fully computerized and participants were instructed to insert their answers in an empty text box. In total, the task lasted for 3 min and was automatically terminated afterward. Responses of the AUT were rated on the creativity, novelty and usefulness dimension by two judges. Judges were instructed to first get an overview of the responses and be in a neutral mood when rating. While rating, scores should be consistent (e.g., same ideas should receive the same score) and their focus should lie only on the respective dimension to be rated. As for creativity, judges should follow their first impression on how creative they perceive the idea to be. For novelty, judges should evaluate the ideas on how novel and unique they are. For usefulness, judges should evaluate the ideas on how well they think the idea will work and can be implemented. Each item in the respective dimension was to be rated on a Likert-scale from 1 to 5, where 1 was the lowest and 5 the highest score (i.e., 1 = not at all creative and 5 = very creative). ICC was calculated with the ICC function of the psych package (Revelle, 2016) in R (R Core Team, 2016). For all rating dimensions, the intraclass correlation (ICC2k) was excellent (*AUTcreativity ICC* = 0.92; *AUTnovelty* *ICC* = 0.87; and *AUTusefulness* *ICC* = 0.94). Accordingly, the average score of the two raters were used for further analysis. *Fluency* was determined by counting the number of answers that were provided.

### Convergent Thinking, the Compound Remote Associate Task (CRAT)

To measure convergent thinking, the German version of the CRAT, validated by Landmann et al. (2014), was used. In the CRAT, participants are asked to find a matching word that relates to three other words previously mentioned. For example, the solution for the triplet “cottage–Swiss–cake” is “cheese.” The CRAT in this study consisted of 20 randomly chosen (and matched by difficulty) triplets using the sample function of the core package in R. All 20 triples were simultaneously shown. Participants had 5 min to solve as many CRAT items as possible. The CRAT was scored according to the solution scheme provided by Landmann et al. (2014). Every response was manually checked for correctness (by the researcher) and the final score was the sum of all correct answers.

### Real-Life Creativity, the Creative Achievement Questionnaire (CAQ)

The CAQ measures real-life creative achievement, and has been validated by Carson et al. (2005). The original English version of the CAQ was administered without a time constraint and included ten categories (with each eight response options) covering different real-life domains. Those domains were: visual arts, music, dance, architectural design, creative writing, humor, inventions, scientific discoveries, theater and film, and culinary arts. All 10 domains were shown at the same time, and response options ranged from 0 to 7, where 7 was the highest score. The CAQ score is the sum of all questions, where in each domain the highest score (if applicable) is multiplied with the amount of times it was fulfilled. For example, in the theater and film domain the most extreme answer is: *“My theatrical work has been recognized in a national publication.*” (7 points). If someone had fulfilled this condition several times (e.g., 3) than the score gets multiplied (i.e., 3 × 7 = 21). The CAQ was scored in accordance to Carson et al. (2005).

### Association Chain Task (ACT)

The ACT is a task designed to capture semantic associations in order to first calculate the mean SmD, and to subsequently derive scaling estimates from its times-series. We used an adapted version from Benedek et al. (2012). Therefore, a customized computer script in python (version 2.7.12), using mainly the PsychoPy package 1.82.1 (Peirce, 2007), was created. The script displayed the instructions and enabled participants to insert written responses on a computer. In the ACT, participants were asked to generate a chain of associations. A definition of associations was provided as follows: “Think of associations as ideas or thoughts between two (or more) concepts.” Accordingly, the task was to repeatedly form an association to the previous word they had generated and continue to do so until the program indicated to stop. As an illustration, let us assume that the starting word was “airplane.” Now the participant had to form an association, for example “vacation,” to the previous word “airplane.” The next association, for example “beach,” had now to be based on the previous word, which is “vacation.” This procedure was now to be repeated. One example has then been displayed to the participant (i.e., apple–tree–leaf–bird), followed by the instruction to only use words which could be found in a dictionary (e.g., no names of friends or actors, book titles, movies, etc.). This was due to the restrictions of the LSA. Then, a practice block of four trials was provided. Hereafter, it was stated that the target block will start. The first word for all participants was “house.” Participants inserted their responses using a keyboard and continued each trial by pressing the “Enter” button. In each trial, only the previously inserted word was shown, together with the instruction to form an association to it. Importantly, it was also mentioned that participants should “try to be creative” in their responses, which was displayed during every trial in the target block. All written responses were recorded within a time window of 35 min. This duration was chosen based on a pilot to maximize the number of trials while considering the tiresome characteristic of the experiment. There were no time restrictions between the trials. That is, participants were free to “think” as long as needed. The task was automatically terminated hereafter.

### LSA and SmD

LSA was used to derive the SmD from the associations formed in the ACT. Conceptually, in LSA a semantic space is created by counting word frequencies of a large body of documents. Accordingly, the first step of LSA is to count the occurrence and co-occurrence of words in documents. That is, each word reflects a row and each document reflects a column in a large matrix. The cells of this matrix are then populated with the number of occurrence of the respective word in that respective document. This matrix is then transformed so that less frequent words have an increased impact, since less frequent words normally convey more detailed and specific meaning, and more frequent words decrease in their impact. Then, a so called, singular value decomposition is applied to the matrix. A method that shares conceptual resemblance with principal component analysis, where factors are formed. Lastly, the dimensions or factors are reduced (mostly to around 300 dimensions) to remove noise and redundant dimensions from the matrix. The result is a (still) highly dimensional semantic space, where words are represented as vectors in this space. The SmD is now defined as the inverted cosine of the angle between two words or vectors in the space. A value of one is interpreted as unrelated words, whereas zero indicates identical words. Thus, words with similar meaning tend to have low values and vice versa. It is possible to receive negative values. However, these values cannot be interpreted and are usually set to one (Deerwester et al., 1990; Landauer et al., 1998; Günther et al., 2015). Subsequently, a mean SmD per participant was calculated to analyze its correlation with the other creativity measures.

### Temporal Structure and Complexity Estimates

The techniques used to infer the temporal structure and to calculate the complexity estimates were all applied in MATLAB. One method to estimate the temporal structure is found in PSD (Gilden et al., 1995). The purpose of PSD is to calculate an estimate of the fractal dimension which informs us about the temporal structure of a time-series (i.e., from random to flexible-stable to persistent). Fractal dimension refers to the presence of self-similar patterns across multilayered scales (see **Figure 1**, left panel). PSD functions most reliable with large times-series consisting of any number that is the power of 2^n^ (e.g., … 1024, 512, 256, 128 …) Conceptually, in PSD a time-series is transformed into a linear combination of sinus waves, called Fourier transformation. The result is a summation of all frequencies and amplitudes of the time-series. All frequencies and amplitudes are log transformed and plotted with (log) frequency on the x-axis and (log) amplitude on the y-axis. The best-fitted line (linear regression) represents the fractal dimension where a slope of -1.0 reflects a (perfect) flexible-stable structure, 0 reflects a (perfect) random structure and -2 reflects a (perfect) persistent structure. The PSD analysis was conducted using the PSD function, available in the Signal Processing Toolbox in MATLAB (The Math Works, 2017).

Detrended fluctuation analysis is another method to inquire the same question in the time domain (Peng et al., 1994). It takes a time-series and computes the cumulative sum. In the present study, the cumulative sum of the SmD time-series is taken. Then, the new time-series of the cumulative sum is divided into several windows with different lengths. For example, a time-series with 100 data points could be divided into 4 (windows) × 25 (length), 5 × 20, 10 × 10, and so on. For each window, a slope (linear regression) is fitted which represents the “local trend.” The “global trend,” which is the regression of the whole time-series, is then subtracted from each “local trend” and hence, detrended. Now, the standard deviation in each window is calculated where the mean is taken and log transformed. Ultimately, the log mean is plotted against the log window sizes and the best fitted line or slope (as in PSD) represents the fractal dimension. A slope of 1 reflects a (perfect) flexible-stable structure, 0.5 reflects a (perfect) random structure and 1.5 reflects a (perfect) persistent structure. The DFA function for MATLAB can be found on https://github.com/FredHasselman/toolboxML/blob/master/Ddfa.m (Schmidt, 2001).

**FIGURE 2:**
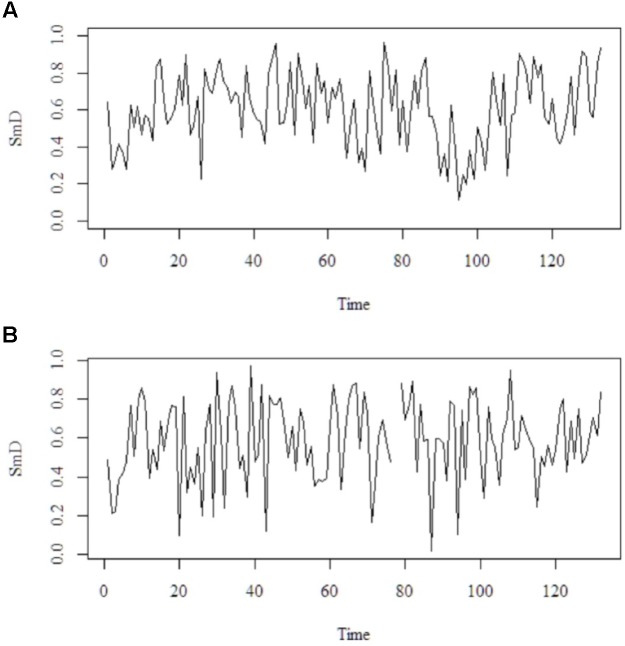
Illustration of change of SmD over time. Two example time-series (raw data) with time on the x-axis and SmD on the y-axis. Upper time-series **(A)** is associated with a PSD = -0.98 and DFA = 0.94. Lower time-series **(B)** is associated with a PSD = -0.20 and DFA = 0.80. Notice that PSD and DFA of **(B)** contradict each other. That is, PSD points at more random pattern but DFA at more flexible-stable pattern.

These methods are complementary in that the strengths of one compensates for the weaknesses of the other. For instance, PSD, while robust in many respects, requires preprocessing of the signal because extreme observations can contaminate the outcome of the analysis (see Holden, 2005). DFA can be applied to non-stationary signals and is not susceptible to most statistical artifacts or long-term trends, but it can falsely classify certain types of signals as fractal (Rangarajan and Ding, 2001). Finally, each participant received a PSD and DFA estimate in addition to the mean SmD to their time-series derived from the ACT (see **Figure 2** for an example of the time-series).

### Procedure

Because of the demanding characteristic of the association task (ACT), it was chosen to split the study in two parts. In part one, an online experiment administered in *Qualtrics*, participants had to complete the AUT, the CRAT, and the CAQ. Moreover, demographic information was assessed. Upon completion of part one, part two, which took place in a lab setting, was scheduled. In the lab study, the ACT was administered and participants had to form a chain of associations. The first word “house” was provided by the program, participants then started to generate associations for 35 min. After the task was finished, participants were thanked, rewarded and debriefed if wished so.

### Data Aggregation

#### LSA and SmD

Before conducting the main analysis, responses of the ACT had to be preprocessed. Therefore, an R script was written in which all responses (10722 words generated by 59 participants) were first cleaned from unwanted characters (i.e., whitespace, special characters, and upper case characters). Hereafter, the SmD was calculated using the Cosine function of the LSAfun package (Günther et al., 2015). The semantic space (“dewak100k_lsa,” a semantic space of the German language) was retrieved from http://www.lingexp.uni-tuebingen.de/z2/LSAspaces/, which was created by the package maintainer. In the first iteration, there were 2627 instances where a SmD could not be calculated. This was mostly due to typos but also due to words (very rare words or compound words) not present in the semantic space. Typos were identified (1624 misspelled words) using the hunspell_check and hunspell_suggest function of the hunspell package (Ooms, 2017). Those words were manually corrected (for a complete list see the **Supplementary Table 1**). In a second iteration, SmDs were newly calculated with the updated data file, which decreased the number of missing values to 857 (8% of all words).

#### Temporal Structure and Complexity Estimates

Before calculating the complexity estimates, the time-series of the SmD for each participant was ensured to be an integer power of 2^n^ (e.g., 64, 128, 256, 512,…). Some algorithms require the length of a time-series to be an integer power of 2. Further the algorithm works faster if this requirement is met. As many time-series did not obey this rule, we used zero-padding to guarantee the length of any time-series to be an integer power of 2. This was done by adding zeros at the end of the time-series. For example, if a time-series had 115 data points, 13 zeros were added at the end to obtain 128 observations (or e.g., 230 + 26 zeros). This, so called zero-padding, is assumed to have no distorting effect on the complexity estimates (Holden, 2005).

## Results

### Main Analysis

To test the main hypothesis that novelty and creativity in the AUT can be modeled as a quadratic function of PSD and DFA, two multiple regressions, one with AUT_novelty_ and the other with AUT_creativity_ as dependent variable and PSD and DFA (and their quadratic transformations) as independent variables were conducted. The regression models for AUT_creativity_ and for AUT_novelty_ were non-significant, *F*(4,52) *=* 2.01, *p* = 0.11, *R^2^* = 0.07 and *F*(4,52) = 1.52, *p* = 0.21, *R^2^* = 0.04, respectively. No linear or quadratic effect were found, and the main hypothesis was not confirmed. Notice that PSD and DFA was not found to be correlated in this study (*r* = -0.03, *p* = 82), which is unusual. For example, in more repetitive tasks the observed correlation were rather high and around *r* = 0.8 (Wijnants et al., 2012). We further discuss this issue in the exploratory analysis section.

### Correlation Between Creativity Measures and Semantic Distance

To assess the bivariate correlation between the AUT, CRAT, CAQ, and SmD, Pearson correlations were calculated (see **Table 2**). The creativity and novelty dimension of the AUT were significantly positively related with each other, usefulness was negatively related to them and to fluency. This is in line with previous research (e.g., Diedrich et al., 2015), in that more useful ideas are usually rated less novel and less creative. On the other side, the more novel an idea is, the more creative it will also be rated. Surprisingly, SmD was not related to the creativity measures, which contradicts previous research (e.g., Prabhakaran et al., 2014; Green, 2016).

**Table 2:** Bivariate correlation of the AUT, CRAT, CAQ, and SmD.

	AUT					
	Creativity	Novelty	Usefulness	Fluency	CRAT	CAQ	SmD
AUT							
Creativity							
Novelty	0.93^∗∗∗^						
Usefulness	-0.28^∗^	-0.40^∗∗^					
Fluency	0.19	0.28^∗^	-0.28^∗^				
CRAT	0.27^∗^	0.20	-0.03	0.14			
CAQ	0.04	0.04	-0.07	-0.02	0.19+		
SmD	0.00	0.02	0.02	0.02	-0.07	0.02	

*N = 58 as one CAQ scores was unreasonable, ^∗^*p* < 0.05, ^∗∗^*p* < 0.01, ^∗∗∗^*p* < 0.001, and +*p* < 0.10*.

### Exploratory Analysis

Previous research successfully made use of SmD as a creativity measure (e.g., Green et al., 2010; Beaty et al., 2014; Prabhakaran et al., 2014). However, the design in these studies was different to the design used in the current study. For example, the experimental duration was approximately 9 min in Prabhakaran et al. (2014) over 72 trials, but 35 min in the present study. To test whether the mean SmD of different task durations (9, 5, and 2 min) would be associated with the creativity measures in this study, bivariate correlations were calculated. All three SmD task durations remained non-significant in relation to AUT_usefulness_, AUT_novelty_, AUT_creativity_, AUT_fluency_, CRAT, and CAQ (all *p* > 0.05). That is, neither was the SmD of the first 9 min related to any creativity measure, nor the SmD of the first 5 or 2 min. These results would strongly argue against SmD as a measure for creativity. However, SmD was significantly predicted by the mean response time in a linear regression *F*(1,57) = 14.38, *p* < 0.001, *R^2^* = 0.20, and β = 0.04, in that longer response time was associated with higher SmD. This is also often described as the serial order effect stating that more creative outcomes tend to appear after increased amount of time (e.g., Christensen et al., 1957; Beaty and Silvia, 2012). This suggest that SmD is likely to reflect the similarity of semantic concepts, as the longer someone thinks, the more uncommon, distinct and hence less similar the response should be.

For the complexity measures, the data showed no significant correlation between PSD and DFA, *r*(57) = -0.03, *p* = 0.83. This is unusual as PSD and DFA are estimating the same relationship and should therefore corroborate each other. A reason could be the variability in the length of the time-series, which varied from as low as 42 to more than 370 data-points. PSD and DFA tend to be more reliable with more observations, while PSD performs best with 2^n^ observations (Delignieres et al., 2006). To introduce a more robust measure, also for smaller time-series, the *sample entropy* was calculated (Richman and Moorman, 2000). The implementation in MATLAB can be found on https://www.physionet.org/physiotools/sampen/matlab/1.1-1/sampenc.m (Lake et al., 2008). Sample entropy is another method to infer the fractal dimension of a time-series, where the higher the sample entropy the more random a system is. However, it is a relative method in that no absolute statements (i.e., time-series A reflects a flexible-stable pattern) can be made. That is, different time-series can be described in their structure to each other in that particular sample (time-series A is more random than B). A bivariate correlation between sample entropy and all creativity measures (AUT, CRAT, and CAQ), mean response time and mean SmD was conducted. Sample entropy did not significantly correlate with AUT_creativity_, *r*(57) = 0.20, *p* = 0.13, or AUT_novelty_, *r*(57) = 0.19 *p* = 0.15^2^. Which further argues against a relationship between the temporal structure of associations and divergent thinking. Interestingly, the correlation between sample entropy and mean SmD was found to be significant, *r*(57) = 0.50, *p* < 0.001. This would indicate that the more random the temporal structure was, the higher the SmD. Although a significant correlation could be detected, caution is advised. There was no predefined hypothesis, and multiple testing inflates type I errors. Moreover, sample entropy was not correlated with PSD, *r*(57) = 0.03, *p* = 1 or DFA, *r*(57) = 0.01, *p* = 1.

## Discussion

This study investigated the temporal structure (i.e., random/flexible-stable/persistent structure) of associations and its relationship to a core component of creativity, divergent thinking. It was hypothesized that novelty and creativity in divergent thinking, as measured by the AUT, would be a quadratic function of the temporal structure of the associations. That is, random and persistent structures of associations were assumed to be related to less novelty and creativity ratings, whereas flexible-stable structures of associations would predict high novelty and creativity ratings on the AUT. The current findings provide no evidence for the hypothesis that the structure of associations is related to an individual’s potential for divergent thinking. There was neither a linear nor quadratic trend found. Initially, this would imply that different structures of associations do not contribute to the ability to generate novel and creative responses. The temporal order of how each association leads to another was irrelevant. Hence, any sort of temporal structure would equally enable people to utter creative behaviors, and a more random structure of associations would be found to display the same relationship with divergent thinking as a persistent structure. If this holds true, it would not matter which structure of associations someone possess, distinct streams of thought would play no role in creativity. This would be in line with Benedek and Neubauer (2013) who found that associative hierarchy is not predictive for divergent thinking (cf. Mednick, 1962).

On the other hand, previous research on semantic properties and creativity were found to corroborate the idea that there might be a relationship (e.g., Forthmann et al., 2016; Hass, 2017a). Furthermore, studies on semantic networks suggest the same, network properties do influence divergent thinking abilities (Kenett et al., 2016, 2017). In a recent study by Kenett and Austerweil (2016), it was tested whether a simulated “search” over modeled semantic networks of more and less creative individuals would lead to different results. Results indicated that, indeed, a simulated “search” in the semantic network of more creative individuals yielded more unique words. Hence, there is growing evidence in the literature that divergent thinking benefits from distinct characteristics in semantic structures. The present study examined the role of the temporal structure of association, which is not fully comparable to associative hierarchy but more relatable to semantic networks. Considering the characteristics of different temporal structures in complex systems (i.e., random, flexible-stable and persistent) one would reason that, e.g., a persistent structure of associations will not facilitate divergent thinking. That is, if every next association heavily builds on the earlier association (e.g., dog–cat–mouse–cheese–etc.) creative thoughts are rare or slowly to appear. Too random structures will presumably not connect concepts meaningful enough. Flexible-stabile structures, in turn, would enable new associations to arise which still form enough coherence to previously generate thoughts (see, e.g., Kenett et al., 2018). If the results are indeed trustworthy, those ideas could be questioned. However, there are reasons to believe that the methodology in the current study did not truly capture the temporal structure, which is laid out in the next paragraph.

### Limitations

#### Temporal Structure of Associations

There are indications that the current results might be less reliable due to noticeable deviations in the data. Firstly, PSD and DFA were not correlated (also not with sample entropy), which could mean that those measures were not capable of estimating the temporal structure of the time-series. One reason might lie in the fluctuation in the number of observations (42 to more than 370) within the time-series and the missing data (8% on average) (Delignieres et al., 2006). Another reason could be the operationalization of associations. It was hypothesized that SmD would capture change in a cognitive process (association formation). PSD and DFA are suggested to reveal natural processes which have been successfully implemented in biologically sound concepts such as heart rate variation (Van Orden et al., 2011) or reaction time (Van Orden et al., 2003). Those processes are outcomes of a natural system. On the other hand, the current research made use of SmD as a proxy for a natural process which was the change of association forming. Because SmD is based on a computational method (LSA), it is likely that it is not an inherently natural and ontologically concise cognitive outcome. When applying techniques to infer the fractal dimension, it is not guaranteed that the result will reflect a true temporal structure of a natural system.

#### SmD and Creativity

SmD was not related to any creativity measure, which strongly contradicts the literature on SmD and creativity (e.g., Beaty et al., 2014; Prabhakaran et al., 2014; Weinberger et al., 2016). Even after considering the greater length of this experiment (previous studies measured SmD in shorter designs), that is, assessing the SmD of the first 9, 5 and 2 min, no relationship with any creativity measure was found. Thus, it is unlikely that the length of the experiment confounded the correlation between SmD and creativity measures. As other studies confirmed the effective application of SmD (e.g., Green et al., 2010; Beaty et al., 2014; Prabhakaran et al., 2014), it is highly likely that another feature of the design in this experiment confounded the effect. For example, the ACT challenged the participants to form associations based on the previous concept. In earlier studies using SmD, associations were to be formed toward one single concept. That is, to form a verb to a noun (Prabhakaran et al., 2014), synonyms to a word (Beaty et al., 2014) or analogies between two words (Weinberger et al., 2016). Notice, however, that reaction time in the ACT significantly predicted SmD in a positive direction (longer reaction time equals greater SmD). Thus, it seems that SmD is related to uncommonness, where the longer someone thinks, the more unusual the response should be (support for internal validity). This is also in line with previous findings, which found that instances of more unusual responses increase over time (Benedek and Neubauer, 2013) and that category switching in divergent thinking tasks was indicated by a higher latency (Acar and Runco, 2017).

Another difference lies in the language of the experiment. All participants were of German nationality and spoke German as their first language. Accordingly, the semantic space of the LSA was based on the German language, whereas earlier studies were conducted in English. However, it is unreasonable, although not impossible, to assume that SmD or similarity between concepts are differently perceived by different nationalities and differently reflected in the LSA.

To conclude, several constraints can be attested to the design of this study. SmD might not capture the cognitive process of association formation. Consequently, analysis methods trying to estimate the temporal structure might fail due to the inappropriateness of the data.

### Future Directions

Future research should, therefore, pursue to refine the methodology for assessing the (temporal) structure of associations. Network science could bring benefits to the researcher seeking to investigate semantic structure and how this relates to divergent thinking and creativity. Additionally, the connection between SmD and creativity should be further explored to concisely pinpoint its relationship. Some authors successfully used LSA to also study sentence-like responses in divergent thinking tasks compared to single-word responses as in this study (e.g., Forthmann et al., 2018). Although this might be possible for LSA and SmD, techniques to infer the temporal structure, such as DFA and PSD, would only yield meaningful results with many more observations (favorably 256 and more) than usually available in common divergent thinking tasks. Another example could be to also study the phonological similarity between words or to apply different computational methods, as LSA is only one of several methods (see, e.g., HAL: Lund and Burgess, 1996) to infer the similarity of semantic concepts (Günther et al., 2015). SmD has the potential to complement established creativity measures (which are mainly subjective) as an objective instrument for assessing creative potential. Therefore, we encourage fellow researchers to venture new and potentially fruitful paths by taking inspiration from other fields.

## Conclusion

As stated in prominent journals (e.g., current Frontiers Research Topic description, special issue Journal of Creative Behavior), the creativity research field could benefit from more interdisciplinary work and a broader range of methodological approaches. Existing creativity research often applies a relatively small number of empirical methodologies. In the current study we integrated methodology from computational linguistics and complex systems into creativity researcher to further enhance our understanding of cognitive creativity. Although the current study does not corroborate the idea that a flexible-stable (vs. random/persistent) temporal structure of associations is related to enhanced performance in divergent thinking, it hopefully challenges fellow researchers to refine the recent methodological developments for assessing the(temporal) structure of associations. Moreover, we hope that the current cross-fertilization of methodological approaches inspires researchers to take advantage of other fields’ ideas and methods. To derive at a theoretically sound cognitive theory of creativity, it is important to integrate research ideas and empirical methods from a variety of disciplines.

## Ethics Statement

This study was carried out in accordance with the recommendations of the Code of Ethics for Research in the Social and Behavioral Sciences involving Human Participants by the Ethiek Commissie Sociale Wetenschappen of the Radboud University Nijmegen. The protocol was approved by the Ethiek Commissie Sociale Wetenschappen. All subjects gave written informed consent in accordance with the Declaration of Helsinki.

## Author Contributions

PW conceived the idea, carried out the experiments, analyzed data, and wrote the manuscript. SR and MW supervised, refined the design, and edited the manuscript. MW also analyzed data.

## Conflict of Interest Statement

The authors declare that the research was conducted in the absence of any commercial or financial relationships that could be construed as a potential conflict of interest.

## Footnotes

1 Notice that some researchers do include an explicit instruction to “be creative” in AUTs (e.g., Forthmann et al., 2016; Harrington, 1975) which has effects on the results of divergent thinking tasks.

2 However, there is the possibility that our sample had not enough power to detect the potentially meaningful correlations as they reside between small and medium effect sizes.
