# Supplementary material for: What Enables Novel Thoughts? The Temporal Structure of Associations and Its Relationship to Divergent Thinking
Source: Front Psychol. 2018 Sep 25;9:1771. doi: 10.3389/fpsyg.2018.01771 (PMC6167455; doi:10.3389/fpsyg.2018.01771)
Supplement: Supplementary file 1 [file Table_1.docx]

Supplementary Material

Article Title

Peng Wang, Maarten L. Wijnants, Simone M. Ritter^*^

*** Correspondence:** Simone M. Ritter : s.ritter@psych.ru.nl

# Supplementary Table

**List of corrected words**

*raw response and correction (in German)*

kaese käse

windung windung

fluestern flüstern

chloroplast chloroplast

buero büro

ueberheblichkeit überheblichkeit

marienkaefer marienkäfer

zuege züge

zigarre

monokel

seekrankheit Seekrankheit

alphabet Alphabet

groesse große

ueberforderung Überforderung

unisex

militaer militär

oelzweig ölzweig

gestaendnis Geständnis

hammer hammer

amboss amboss

sanftheit

matraze Matratze

flaeche fläche

durchhaltevermoegen Durchhaltevermögen

menschen kennenlernen menschen kennenlernen

gebraeuche gebräuche

goetter götter

ruecksicht rücksicht

alte menschen alte menschen

gewuerze gewürze

faecher fächer

loewenzahn Löwenzahn

stiel

duenn dünn

essstoerung essstörung

melanin Melanin

wueste wüste

sehenswuerdigkeiten Sehenswürdigkeiten

freheitsstatue Freiheitsstatue

beruehmt berühmt

versoehnen versöhnen

fast food Fastfood

pruefung Prüfung

hochesig hochnäsig

polytheismus Polytheismus

goetter götter

gebaeude gebäude

jongleur

baelle bälle

taeter täter

fruehling frühling

meinungsaeusserung meinungsäußerung

blaetter blätter

baeume bäume

blaubluetig blaublütig

sandburg
